# Kaposi’s sarcoma-associated herpesvirus (KSHV) LANA prevents KSHV episomes from degradation

**DOI:** 10.1128/jvi.01268-23

**Published:** 2024-01-19

**Authors:** Ken-ichi Nakajima, Tomoki Inagaki, Jonna Magdallene Espera, Yoshihiro Izumiya

**Affiliations:** 1 Department of Dermatology, School of Medicine, University of California Davis, Sacramento, California, USA; 2 Department of Biochemistry and Molecular Medicine, School of Medicine, University of California Davis, Sacramento, California, USA; Lerner Research Institute, Cleveland Clinic, Cleveland, Ohio, USA

**Keywords:** KSHV, latency, episome, protein knockdown

## Abstract

Protein knockdown with an inducible degradation system is a powerful tool for studying proteins of interest in living cells. Here, we adopted the auxin-inducible degron (AID) approach to detail Kaposi’s sarcoma-associated herpesvirus (KSHV) latency-associated nuclear antigen (LANA) function in latency maintenance and inducible viral lytic gene expression. We fused the mini-auxin-inducible degron (mAID) tag at the LANA N-terminus with KSHV bacterial artificial chromosome 16 recombination, and iSLK cells were stably infected with the recombinant KSHV encoding mAID-LANA. Incubation with 5-phenyl-indole-3-acetic acid, a derivative of natural auxin, rapidly degraded LANA within 1.5 h. In contrast to our hypothesis, depletion of LANA alone did not trigger lytic reactivation but rather decreased inducible lytic gene expression when we stimulated reactivation with a combination of ORF50 protein expression and sodium butyrate. Decreased overall lytic gene induction seemed to be associated with a rapid loss of KSHV genomes in the absence of LANA. The rapid loss of viral genomic DNA was blocked by a lysosomal inhibitor, chloroquine. Furthermore, siRNA-mediated knockdown of cellular innate immune proteins, cyclic AMP-GMP synthase (cGAS) and simulator of interferon genes (STING), and other autophagy-related genes rescued the degradation of viral genomic DNA upon LANA depletion. Reduction of the viral genome was not observed in 293FT cells that lack the expression of cGAS. These results suggest that LANA actively prevents viral genomic DNA from sensing by cGAS-STING signaling axis, adding novel insights into the role of LANA in latent genome maintenance.

**IMPORTANCE:**

Sensing of pathogens’ components is a fundamental cellular immune response. Pathogens have therefore evolved strategies to evade such cellular immune responses. KSHV LANA is a multifunctional protein and plays an essential role in maintaining the latent infection by tethering viral genomic DNA to the host chromosome. We adopted the inducible protein knockdown approach and found that depletion of LANA induced rapid degradation of viral genomic DNA, which is mediated by innate immune DNA sensors and autophagy pathway. These observations suggest that LANA may play a role in hiding KSHV episome from innate immune DNA sensors. Our study thus provides new insights into the role of LANA in latency maintenance.

## INTRODUCTION

Kaposi’s sarcoma-associated herpesvirus (KSHV) is an etiological agent of Kaposi’s sarcoma (KS), primary effusion lymphoma, multicentric Castleman’s disease, and KSHV inflammatory cytokine syndrome (1). KSHV is one of a few pathogens recognized as a direct carcinogen, which also includes hepatitis B virus, human papillomavirus, and Epstein-Barr virus (2–6). Significant effort has been made to help patients by finding a cure for devastating diseases, but we have not been successful yet. Better understanding of essential viral proteins for the KSHV life cycle is therefore critically important.

Like other herpesviruses, KSHV exhibits a biphasic life cycle consisting of a life-long latent infection phase and a transient lytic reactivation phase, which are distinguished by their gene expression profiles (7–10). During the latent phase, the KSHV genomic DNA persists as a circular episome in the host cell’s nucleus (11–14), and the majority of viral gene expression is silenced (14–18). Upon reactivation from latency, the full repertoire of inducible viral genes is activated in a temporally regulated manner, leading to the transcriptional activation of three classes of lytic genes, referred to as immediate early, early, and late genes (8, 10). ORF73 is one of the few genes expressed in the latent phase and encodes the latency-associated nuclear antigen (LANA). Previous studies have shown that LANA is necessary for the persistence of the viral genome in an episomal state. LANA directly binds to the conserved terminal repeat (TR) sequences in the KSHV genome through its C-terminal domain and docks onto the host chromosome through its N-terminal chromatin-binding domain, thus allowing the viral genome to hitch a ride on the host chromosome during mitosis and maintain stable episomal copy numbers in the latently infected cells (19, 20). The presence of LANA is sufficient to mediate the replication and maintenance of a plasmid containing the KSHV latent origin of replication (ori-P) in transfected cells (21, 22). LANA tethers KSHV epsiomes by interacting with cellular histone H2A and H2B and also recruits cellular DNA replication machinery to TRs during the S-phase of the cell cycle (23). LANA also forms a complex with many histone-associated proteins such as bromodomain-containing proteins 2 and 4, KDM3A, polycomb repressor complex 2, hSET1 complex, chromodomain helicase DNA binding protein 4, and MLL1 complex (24–32). It has been proposed that LANA antagonizes the transcription of lytic genes during latency and facilitates the establishment of latent infection via multiple mechanisms. For example, LANA inhibits K-Rta expression, a viral transactivator essential for lytic reactivation, by repressing the transcriptional activity of the K-Rta promoter (33). Another mechanism includes recruitment and tethering of PRC2, CTCF, and cohesin (8, 34–36). These studies primarily utilize gene knockdown or knockout that may introduce indirect effects that compensate the loss of LANA biological activity during the establishment of the cell clones.

RNA interference (RNAi)-mediated gene silencing and CRISPR/Cas-mediated gene knockout have been utilized widely to study the function of a specific gene/protein of interest. However, some genes are essential for cell growth or survival, and those genes are difficult to be silenced transcriptionally or knocked out. In contrast, inducible protein degradation, also known as protein knockdown, is a versatile approach for studying the function of a specific gene/protein that is essential for cell growth (37). The auxin-inducible degron (AID) system has recently emerged as a powerful tool to conditionally deplete a target protein in various organisms, and the AID-tagged target protein can be degraded within a few minutes to a few hours after the addition of the plant hormone auxin (38, 39). Mechanistically, rice-derived TIR1 (OsTIR1) protein interacts with endogenous Skp1 and Cul1 proteins to form a functional Skp1-Cul1-F-box (SCF) E3 ubiquitin ligase complex in non-plant cells [see reference (38)]. The OsTIR1-containing SCF E3 ubiquitin ligase is activated only when the plant hormone auxin [or its derivatives such as 5-phenyl-indole-3-acetic acid (5-Ph-IAA)] is present. The target protein will then be polyubiquitinated and degraded by the proteasome. Because the knockdown of proteins through inhibition of transcription would take some time, cells often adapt to the changes by compensating for the biological effects through other means. In contrast, rapid protein depletion has bypassed these indirect and undesired effects and therefore has allowed researchers to identify more direct biological effects of the proteins of interest.

Cellular innate immunity is the first line of defense against incoming pathogens, including bacteria and viruses. The innate immune system is an evolutionally conserved host defense with key features being shared between plants and animals (40, 41). Several fundamental molecules/mechanisms for cellular innate immunity responses have been identified, including pattern recognition receptor (PRR) signaling [e.g., toll-like receptors (TLRs), nucleotide oligomerization domain-like receptors, and RIG-I-like receptors] (42) and a DNA sensor cyclic AMP-GMP synthase (cGAS) (43). PRRs recognize microbial components known as pathogen-associated molecular patterns (42). Examples of these include bacterial cell wall components such as lipopolysaccharides and ribonucleic acids (RNA) derived from viruses. PRRs recognize and bind their respective ligands and recruit adaptor molecules through their effector domains and then initiate downstream signaling pathways to exert effects. On the other hand, cGAS has recently emerged as a non-redundant DNA sensor invaluable for detecting many pathogens (43). The binding of cGAS to double-stranded DNA (dsDNA) allosterically activates its catalytic activity and leads to the production of 2′3′ cyclic GMP-AMP (2′3′-cGAMP) from ATP and GTP, which acts as a second messenger and potent agonist of the downstream protein, simulator of interferon genes (STING). For this reason, cGAS can recognize a broad range of DNA species of both foreign and self-origin. Binding to 2′3′-cGAMP triggers conformational changes in STING, followed by its trafficking from the endoplasmic reticulum (ER) to the Golgi apparatus via the endoplasmic reticulum-Golgi intermediate compartment (ERGIC) (43). Upon reaching the ERGIC/Golgi, STING is oligomerized into a signaling platform that recruits downstream proteins to initiate an immune response, including cytokine production (43). In addition, a recent study revealed that STING also induced the autophagy pathway to degrade components derived from pathogens as a cellular defense response (44).

In this study, we constructed a KSHV bacterial artificial chromosome 16 (BAC16), in which the N-terminus of LANA is tagged with a 7 kDa mini-auxin-inducible degron (mAID) tag. We assessed the contribution of LANA for maintaining viral episomes in infected cells and inducible lytic gene regulation during reactivation.

## RESULTS

### Generation and characterization of mAID-LANA BAC16

**Fig 1:**
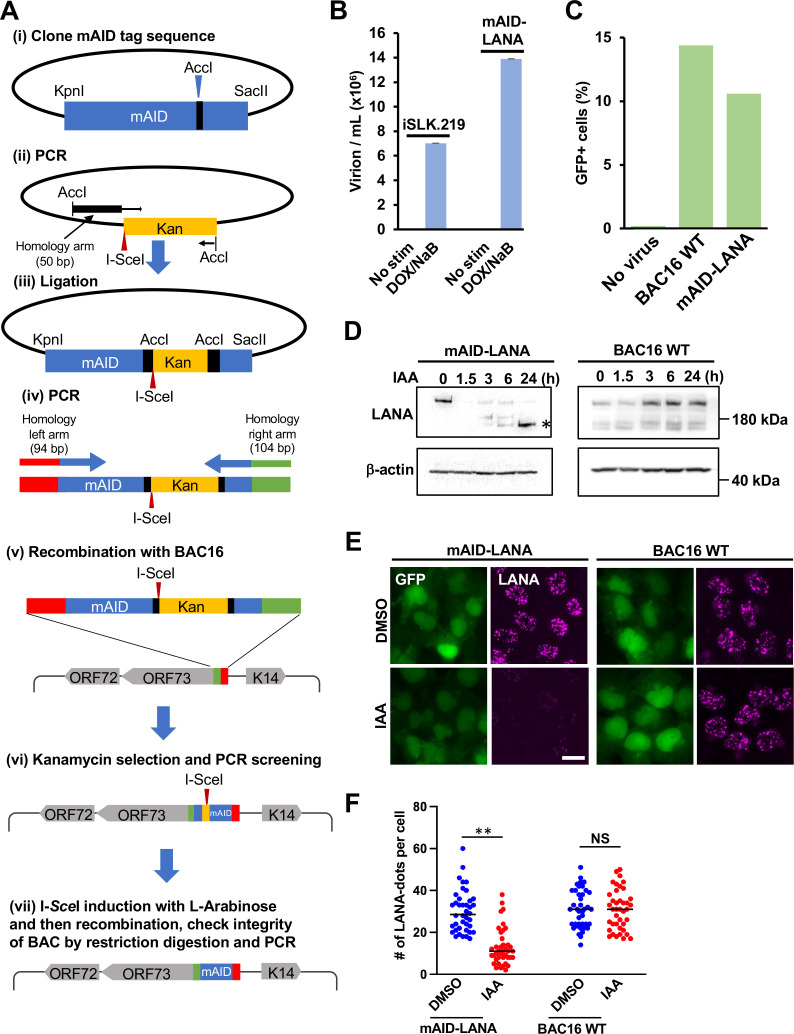
Generation of mAID-LANA BAC16. (**A**) The schematic diagram for construction of mAID-LANA with KSHV BAC16. (i) The codon-optimized cDNA fragment for mAID (mAID KpnI-SacII pBS fragment) was synthesized and cloned into the pBlueScript vector between the KpnI and SacII restriction enzyme sites. (ii) The kanamycin cassette with I-SceI recognition sequence, along with 50 bp of the homologous sequence, was generated by PCR with pEP-Kan plasmid as a template and mAID-SacII fragment KanFw and mAID-SacII fragment KanRv as primers, and cloned into the AccI restriction enzyme site. (iii–v) The resulting plasmid was fully sequenced and used as a template to generate a DNA fragment for homologous recombination with BAC16 inside bacteria. mAID-ORF73N-Fw and mAID-ORF73N-Rv were used as primers. (vi and vii) After confirmation of insertion at the correct site by colony PCR screening, the kanamycin cassette was deleted by recombination with induction of I-SceI in bacteria by incubation with L-arabinose. Correct insertion of the mAID-tag and integrity of BAC DNA were confirmed by sequencing of the PCR-amplified fragment and restriction digestions. Primers and the DNA fragments used are listed in Table 1. (**B**) Production of progeny virus. Capsidated viral DNA copy number was quantified by quantitative PCR at 96 h post-stimulation. (**C**) *De novo* infection. iSLK cells were infected with the purified virus at a multiplicity of infection of 10, and the green fluorescent protein (GFP)-positive cell population was quantified by flow cytometry 48 h after infection. (**D**) Depletion of mAID-LANA by 5-Ph-IAA treatment. iSLK-OsTIR1-mAID-LANA BAC16 cells and iSLK-OsTIR1-BAC16 wild-type (WT) cells were treated with 2 μM 5-Ph-IAA [indole-3-acetic acid (IAA)], and LANA depletion was assessed by Western blotting. Asterisks indicate possible degradation products. (**E**) Fluorescence micrographs of cells treated with 2-μM 5-Ph-IAA (IAA) or dimethyl sulfoxide (DMSO) for 24 h. LANA was visualized by immunofluorescence staining. Bar, 20 μm. (**F**) Quantification of LANA-dots. The number of LANA-dots per nucleus was manually counted. **P < 0.01. NS, not significant.

Protein knockdown with the degron system is an effective approach to dissecting the function of proteins of interest in living cells. The protein knockdown is especially suitable for proteins that are difficult to establish stable knockdown or knockout from cells. LANA is an abundantly expressed protein in KSHV latently infected cells and plays an invaluable role in maintaining latency by at least two molecular mechanisms: (i) tethering KSHV DNA to host chromosomes to maintain viral genomes (21) and (ii) suppressing inducible lytic gene expression (45). To reveal LANA’s contribution to latency maintenance, we adapted the inducible protein degradation approach to a recombinant KSHV BAC system and established recombinant KSHV which encodes mAID-fused LANA (mAID-LANA). We first prepared a template plasmid which is used to generate PCR fragments for recombination. The template plasmid contains a kanamycin resistance cassette in the mAID coding sequence as an excisable format with I-SceI induction. The mAID-kanamycin DNA fragment was first amplified with primers with ~100-bp homology arms, and amplified fragments were then used for recombination by using a two-step recombination method as previously described (46–48) (Fig. 1A). The recombination junction and adjacent regions were amplified by PCR, and the PCR fragments were directly sequenced to confirm in-frame insertion. The mAID-LANA BAC16 was transfected into iSLK cells to generate iSLK cells harboring mAID-LANA BAC16.

**TABLE 1:** Primers and DNA fragments used in this study

Primer or gene fragment	Sequence (5′–3′)^*a*^
mAID-SacII Fragment KanFw	AAGCCGCGGCATTCGTAAAAGTCTCTATGGACGGTGCCCCCTACCTTCGGAAAATTAGGGATAACAGGGTAATCG
mAID-SacII Fragment KanRv	ATGCCGCGGGCCAGTGTTACAACCAATTAACC
mAID KpnI-SacII pBS fragment	TAGGGCGAATTGGAGCTCCAGGCGGAGGTGGTTCCAAGGAAAAGTCAGCGTGTCCTAAAGATCCTGCTAAACCT CCTGCCAAAGCACAAGTAGTTGGATGGCCTCCGGTAAGAAGCTATCGAAAGAACGTGATGGTGTCATGTCAAAAA TCAAGCGGCGGACCCGAAGCCGCGGCATTCGTAAAAGTCTCTATGGACGGTGCCCCCTACCTTCGGAAAATAGAC TTGAGAATGTATAAGTAAGGTACCCAGCTTTTGTTCCCTTTAG
mAID-ORF73N-Fw	*CCCTTGTGGTCACTACGGGTATTGCATAATGTGAATATACTGCCACCGCCTCCATAATTTTACTTTGGTTGTCAGACC* *AGATTTCCCGAGGATG* **AAGGAAAAGTCAGCGTGTCCTAAAG**
mAID-ORF73N-Rv	*CCAAGGTCACATCTTTCCGGAGACCTGTTTCGTTTCCTACAACTTCCTCTCGTTAAGGGCGCGCCGGTGCTCCGTC* *CCGACCTCAGGCGCATTCCCGGGGGCGCGCTACCTCCGCCACCGCTACCTCCGCCACCGCTACCTCCGCCACC* **C** **TTATACATTCTCAAGTCTATTTTC**

^*a*^ Restriction enzyme sites used for cloning are underlined. Italics indicate homology arms for recombination. Boldface indicates sequence annealed to cloned mAID coding sequence for amplification DNA fragment for recombination. (GGGGS)×3 linker sequence in between mAID and LANA is italicized with underline.

Next, the cells were transduced with a recombinant lentivirus expressing FLAG-tagged rice-derived TIR1 F74G (FLAG-OsTIR1 F74G) protein. We named this cell subline as iSLK-OsTIR1-mAID-LANA BAC16 cell. OsTIR1 is a high-affinity auxin-binding protein that interacts with endogenous Skp1 and Cul1 proteins to form a functional SCF E3 ubiquitin ligase complex (38). The OsTIR1-containing SCF E3 ligase is only activated in the presence of plant hormone auxin or its derivatives and polyubiquitinates AID-tagged (or mAID-tagged) target protein, which leads to rapid proteasome-mediated degradation. We next verified the virological function of mAID-LANA. We first compared the amount of KSHV virion productions from iSLK-OsTIR1-mAID-LANA BAC16 cells with that from iSLK.219 cells (Fig. 1B). We also examined the infectivity of progeny virions produced from iSLK-OsTIR1-mAID-LANA BAC16 cells. For that, iSLK cells were infected with purified mAID-LANA BAC16 or BAC16 wild-type (WT) virions at a multiplicity of infection (MOI) of 10, and green fluorescent protein (GFP)-positive iSLK cells were quantified by flow cytometry. The GFP-positive (infection) ratio was comparable with that of the BAC16 WT virus (Fig. 1C). Furthermore, we confirmed that KSHV episomes were maintained during cell passage. These results suggested that the tagging with the 68-amino acid residue mAID at the N-terminus did not interfere with the LANA function.

### Rapid degradation of LANA with 5-Ph-IAA

Next, we examined how quickly and to what degree incubation with auxin can deplete LANA. iSLK-OsTIR1-mAID-LANA BAC16 cells were incubated with 2-μM 5-Ph-IAA, a derivative of the natural auxin indole-3-acetic acid, for 0, 1.5, 3.0, 6.0, and 24.0 h. The cells were lysed, and an equal amount of protein was subjected to SDS-PAGE followed by Western blotting with an anti-LANA antibody. As shown in Fig. 1D, LANA was quickly depleted within 1.5 h of incubation with 5-Ph-IAA. In contrast, the expression level of LANA was not changed in response to 5-Ph-IAA in iSLK-OsTIR1-BAC16 WT cells, which harbored wild-type BAC16, indicating that the LANA depletion in response to 5-Ph-IAA is AID system dependent. LANA directly binds to the TR region of viral genomic DNA as multimers and binding to the 30–40 copies of the TR sequences concentrates LANA on the viral genome that makes them visible as “LANA-dots” or “LANA-speckles” (49) (Fig. 1E). Thus, a LANA-dot also indicates a single viral episome in the nucleus (21). As expected, the LANA-dot signal was drastically reduced at 24 h after the addition of 5-Ph-IAA (Fig. 1E and F). We counted the number of LANA-dots, and the result shows that the number of LANA-dots per cell was also significantly decreased by 5-Ph-IAA treatment in addition to intensity of LANA staining (Fig. 1F). In addition, the GFP fluorescence signal was decreased at 24 h after addition of 5-Ph-IAA (Fig. 1E). These results indicated that depletion of LANA might lead to elimination of viral episome in the cell. Consistent with Fig. 1D, neither LANA- signal intensities nor the number of LANA-dots was affected by 5-Ph-IAA treatment in controls (Fig. 1E and F).

### Viral lytic gene expression after depletion of LANA

**Fig 2:**
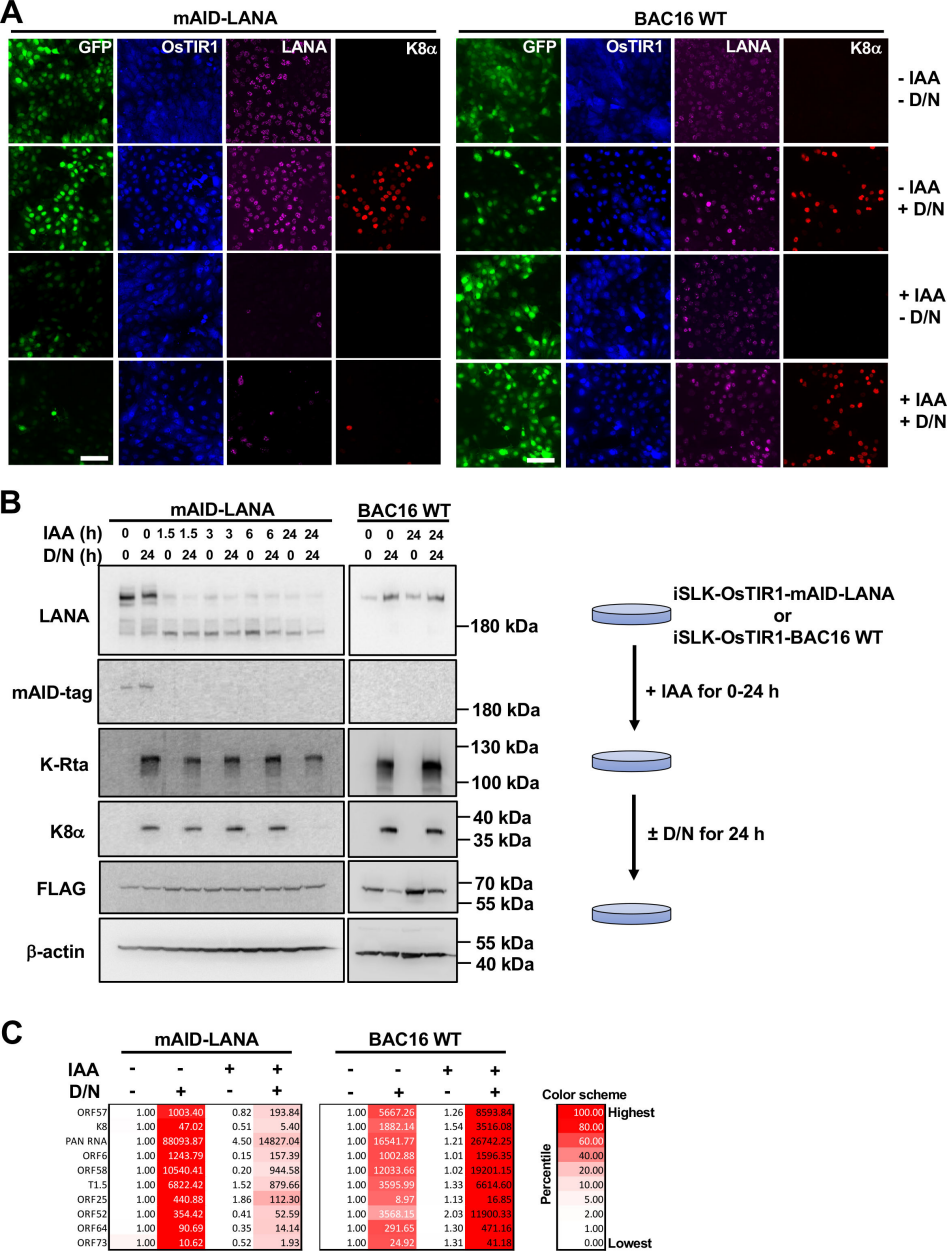
Lytic reactivation after depletion of LANA. (**A**) Immunofluorescence microscopy. iSLK-OsTIR1-mAID-LANA BAC16 cells and iSLK-OsTIR1-BAC16 WT cells were treated with or without 2 μM 5-Ph-IAA (± IAA) for 24 h, treated with or without 1-μg/mL doxycycline plus 1.5-mM sodium butyrate (±D/N) for another 24 h, and then subjected to immunofluorescence staining with anti-FLAG, LANA, and K8α antibodies. Bar, 100 μm. (**B**) Western blotting. iSLK-OsTIR1-mAID-LANA BAC16 cells and iSLK-OsTIR1-BAC16 WT cells were treated with 2-μM 5-Ph-IAA (IAA) for 0, 1.5, 3.0, 6.0, and 24.0 h, and then treated with or without 1-μg/mL doxycycline plus 1.5-mM sodium butyrate (±D/N) for an additional 24 h. The total cell lysate was prepared and subjected to immunoblotting with indicated antibodies. (**C**) Real-time qPCR. Total RNA was extracted and subjected to real-time qPCR. 18S rRNA was used as an internal standard for normalization, and the IAA (−) D/N (−) was set as 1.

We next examined the effects on gene transcription to assess the gene silencing function of LANA. We reactivated iSLK-OsTIR1-mAID-LANA BAC16 cells after the depletion of LANA, and the cells were fixed and stained to visualize viral lytic protein expression. The cells were first treated with or without 5-Ph-IAA for 24 h and reactivated with doxycycline and sodium butyrate for another 24 h. Fluorescence of K8α was seen in approximately 40% of cells without depletion of LANA (the second row in Fig. 2A), and this observation is consistent with previous studies, in which approximately 30%–40% of cells expressed early gene products such as ORF6 (47). To our surprise, the depletion of LANA itself did not globally induce lytic reactivation (the third row in Fig. 2A) but rather inhibited expression of K8α (the bottom row in Fig. 2A) when lytic reactivation was induced with doxycycline and sodium butyrate. Viral lytic gene expression was also confirmed by Western blotting (Fig. 2B) and real time-quantitative PCR (qPCR) (Fig. 2C). Expression of K8α was still seen at 1.5, 3.0, and 6.0 h after the addition of 5-Ph-IAA compared to the control when cells were reactivated. Consistent with the immunofluorescence study (Fig. 2A), K8α expression was substantially decreased at the 24-h time point (Fig. 2B). mRNA expression for lytic genes was decreased to less than one-fourth to one-tenth of control levels at 24 h in LANA-depleted cells (Fig. 2C). However, two highly inducible non-coding RNAs (PAN RNA and T1.5) showed slight increase in the absence of LANA [Fig. 2C, 5-Ph-IAA (+) Dox/NaB (−)] even though many of the other lytic ORF expressions were decreased. The results suggested that LANA depletion alone could activate transcription at limited KSHV genomic domains. As expected, the expression of viral lytic proteins was not affected by 5-Ph-IAA treatment in iSLK-OsTIR1-BAC16 WT cells (Fig. 2A and B), while treatment with 5-Ph-IAA alone slightly increased lytic gene expression in iSLK-OsTIR1-BAC16 WT cells (Fig. 2C).

### Viral episome degradation upon depletion of LANA

**Fig 3:**
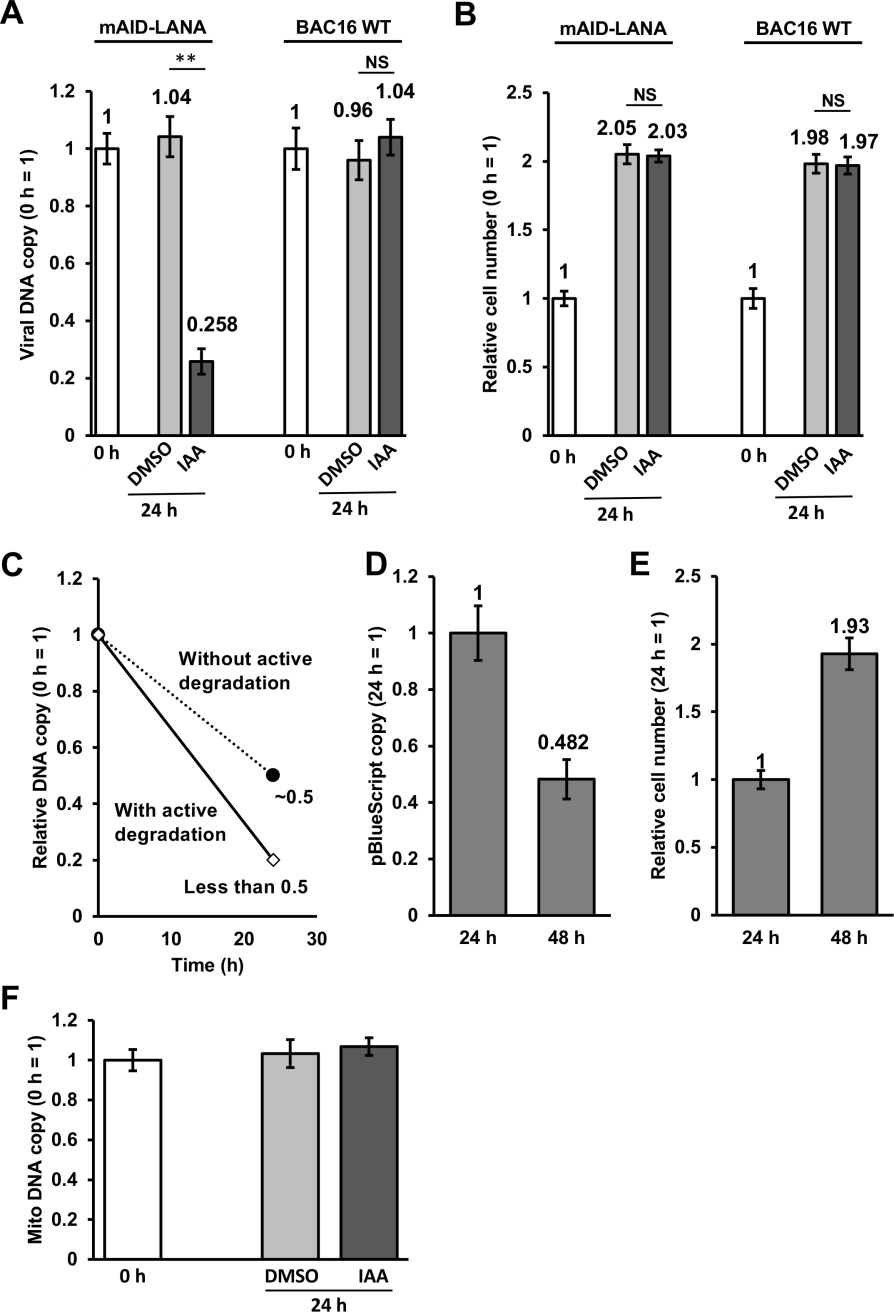
(**A and B**) Rapid reduction of viral DNA copy after depletion of LANA. iSLK-OsTIR1-mAID-LANA BAC16 cells and iSLK-OsTIR1-BAC16 WT cells were treated with DMSO (0.1%) or 2 μM of 5-Ph-IAA (IAA) for 24 h, and then followed by the indicated analyses. (**A**) Total DNA was extracted, and viral DNA copy was measured by real-time qPCR. 18S rRNA was used as an internal standard for normalization. **P < 0.01. (**B**) Cells were trypsinized and suspended in 1% bovine serum albumin (BSA), 0.1-mM EDTA in phosphate-buffered saline (PBS). The number of cells was manually counted by using a hemocytometer. The number of cells at 0 h was set as 1. (**C and D**) Copy number of pBlueScript vector in iSLK-OsTIR1-mAID-LANA BAC16 cells. The cells were transfected with 2 μg of pBlueScript KS+ with Lipofectamine 2000 reagent, incubated for 24 and 48 h, and followed by the indicated analyses. (**C**) Cells were trypsinized and treated with DNase I in order to digest DNA that was present outside of the cells. Total DNA was extracted, and a copy of pBlueScript was measured by real-time qPCR. We used a primer pair for β-lactamase gene encoded by the pBlueScript vector. 18S rRNA was used as an internal standard for normalization. (**D**) Cells were trypsinized and suspended in 1% BSA, 0.1-mM EDTA in PBS. (**E**) The number of cells was manually counted by using a hemocytometer. The number of cells at 24 h was set as 1. (**F**) Copy number of mitochondria DNA in iSLK-OsTIR1-mAID-LANA BAC16 cells. The cells were treated with 2-μM 5-Ph-IAA (IAA) or DMSO for 24 h. Total DNA was extracted, and a copy of mitochondria DNA was measured by real-time qPCR. We used a primer pair for tRNA Leu(UUR) gene encoded by mitochondria DNA. NS, not significant.

Treatment with 5-Ph-IAA reduced LANA-dots signal as well as GFP fluorescence signal in the cells (Fig. 1E and F). The results suggested that depletion of LANA may decrease viral episomal copies in the cell. Accordingly, we next determined KSHV genomic copy number per cell by normalizing it to the host genome. The results showed that the depletion of LANA rapidly reduced viral genomic DNA to approximately 25% of control within 24 h (Fig. 3A). The results were somewhat surprising since iSLK cells are unlikely to divide twice within 24 h. If failure of episome tethering is the only reason for the episome loss, we speculate that having one-fourth of episomes would require at least two cell divisions. We therefore counted the number of cells in the same condition, and the result shows that the cell number only doubled after 24 h (Fig. 3B). We depicted a theoretical graph showing putative DNA copy dilution at 24 h with or without active DNA degradation, when cells divide once in 24 h (Fig. 3C). We also performed the same experiments using iSLK-OsTIR1-BAC16 WT cells as a control. The results showed that (i) these cells also divided once in 24 h, and (ii) KSHV genomic copy number per cell was not significantly changed with 5-Ph-IAA for 24 h (Fig. 3A and B). These observations collectively suggested that active viral episome degradation might be triggered in the absence of LANA.

We next asked if the DNA degradation was specific to the viral episome. We transfected pBlueScript KS+ plasmid into iSLK-OsTIR1-mAID-LANA BAC16 cells, extracted DNA from the transfected cells at 24 and 48 h after transfection, and quantified the copy of pBlueScript plasmid. If the LANA degradation globally increases DNA degradation, we expect a similar decline in copy number. In addition, we also quantified the copy number of endogenous DNA (mitochondria DNA) as a control. As shown in Fig. 3D, pBlueScript copy number at 48 h was 0.48, when we set the copy number at 24 h as 1. We also counted the cell number in the same condition, and the result showed that the relative cell number at 48 h was 1.9 when we set the cell number at 24 h as 1 (Fig. 3E). The copy number of mitochondria DNA was also not changed by 5-Ph-IAA treatment. The results suggested that the depletion of LANA triggered KSHV episome degradation in iSLK cells.

### Viral episome degradation in the lysosome via autophagy

Next, we examined how viral episomes were degraded upon LANA depletion. Lysosome is an organelle in which various endogenous molecules, as well as exogenous molecules, are degraded (50). Particularly, the lysosome plays a crucial role in autophagy-mediated degradation (51). The pH of the lysosomal lumen is kept at 4.5–5.0, while the cytosolic pH is approximately 7.2–7.5 (52). The lysosomal luminal acidic pH is critical for hydrolases localized in the lumen (e.g., proteases and the lysosomal DNase II) that decompose many molecules, organelles, and pathogens and their constituents (50). Chloroquine is a lysosomotropic agent that was historically developed as an anti-malarial drug (53). Chloroquine passively diffuses into the lysosome, where it accumulates as a protonated form. This results in an increase of the intra-lysosomal pH, hence the inhibition of lysosomal hydrolases that require an acidic pH for their proper function (54).

**Fig 4:**
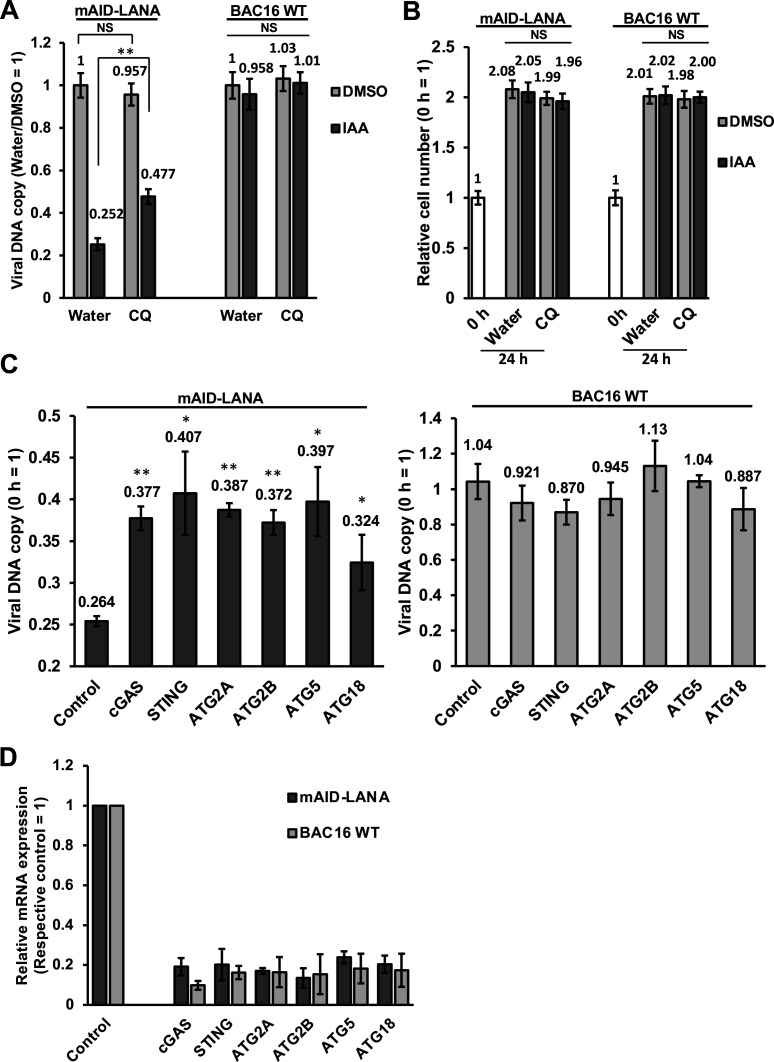
Viral DNA degradation is mediated by cGAS-STING and autophagy pathway. (**A**) Treatment with chloroquine rescued the reduction of viral DNA upon LANA depletion. iSLK-OsTIR1-mAID-LANA BAC16 cells were treated with 50 μM of chloroquine (CQ) for 30 min, and then 5-Ph-IAA (IAA) was added to a final concentration of 2 μM. At 24 h after the addition of 5-Ph-IAA, total DNA was extracted, and viral DNA was quantified as described in the figure legend of Fig. 2. **P < 0.01. (**B**) Relative cell number after treatment with or without chloroquine. The number of cells was manually counted by using a hemocytometer. The number of cells at 0 h was set as 1. (**C and D**) Knockdown of cGAS, STING, and autophagy-related molecules rescued the reduction of viral DNA upon LANA depletion. iSLK-OsTIR1-mAID-LANA BAC16 cells were transfected with 25-nM siRNA against indicated molecules for 48 h. The cells were then treated with 2 μM of 5-Ph-IAA for another 24 h and followed by the indicated analyses. (**C**) Total DNA was extracted, and viral DNA was quantified as described above. *P < 0.05 vs control, **P < 0.01 vs control. (**D**) Total RNA was extracted, and mRNA expression of target genes was quantified by real-time qPCR. Glyceraldehyde 3-phosphate dehydrogenase (GAPDH) was used as an internal standard for normalization. **P < 0.01. NS, not significant.

We first treated the iSLK-OsTIR1-mAID-LANA BAC16 cells with chloroquine for 30 min, and then 5-Ph-IAA was added. The cells were further incubated for 24 h in the presence of chloroquine and 5-Ph-IAA. If viral DNA is degraded in lysosome upon LANA depletion, chloroquine treatment would rescue, and the viral DNA copy should be approximately 0.5 when the cells divided once in 24 h. As shown in Fig. 4A, viral DNA copy was recovered to 0.477 from 0.252 by chloroquine treatment. Note that chloroquine treatment itself did not have effects on viral DNA copy number. We also counted the number of cells in the same condition, and results showed that these cells still divided once in 24 h (Fig. 4B). In addition, we also examined viral DNA copy and cell number of iSLK-OsTIR1-BAC16 WT control cells, and the results showed that neither 5-Ph-IAA nor chloroquine had significant effects on both viral DNA copy and cell number. These results suggest that viral DNA was delivered to the lysosome for degradation in the absence of LANA.

### cGAS is required for the viral DNA reduction

cGAS is an invaluable DNA sensor for cellular innate immunity and plays an important role in the detection of dsDNA derived from pathogens (43). Upon binding to dsDNA, cGAS produces a second messenger 2′3′-cGAMP that activates the downstream protein, STING localized on the ER membrane. STING then migrates from the ER to the ERGIC and/or Golgi apparatus where it stimulates the interferon production (55). In addition to interferon production, a recent study revealed that STING induces autophagy (44). We therefore asked if viral DNA is degraded by cGAS-STING-mediated autophagy when LANA is depleted. We transfected siRNA against cGAS, STING, and several autophagy-related genes (e.g., ATG2A) to see if the knocking down of these genes rescues the viral DNA reduction upon LANA depletion. We first confirmed the gene silencing, and the results showed that siRNAs efficiently reduced targeted mRNA expression in iSLK-OsTIR1-mAID-LANA BAC16 cells. The knockdown of cGAS, STING, ATG2A, ATG2B, ATG5, and ATG18 partially but significantly rescued the viral DNA reduction upon LANA depletion (Fig. 4C and D). On the other hand, the knocking down of these genes itself did not have significant effects on viral DNA copy in the presence of LANA (iSLK-OsTIR1-BAC16 WT cells), suggesting that viral DNA was specifically degraded by LANA depletion (Fig. 4C and D).

### KSHV episome is more stable in the absence of cGAS in human embryonic kidney 293 cells

**Fig 5:**
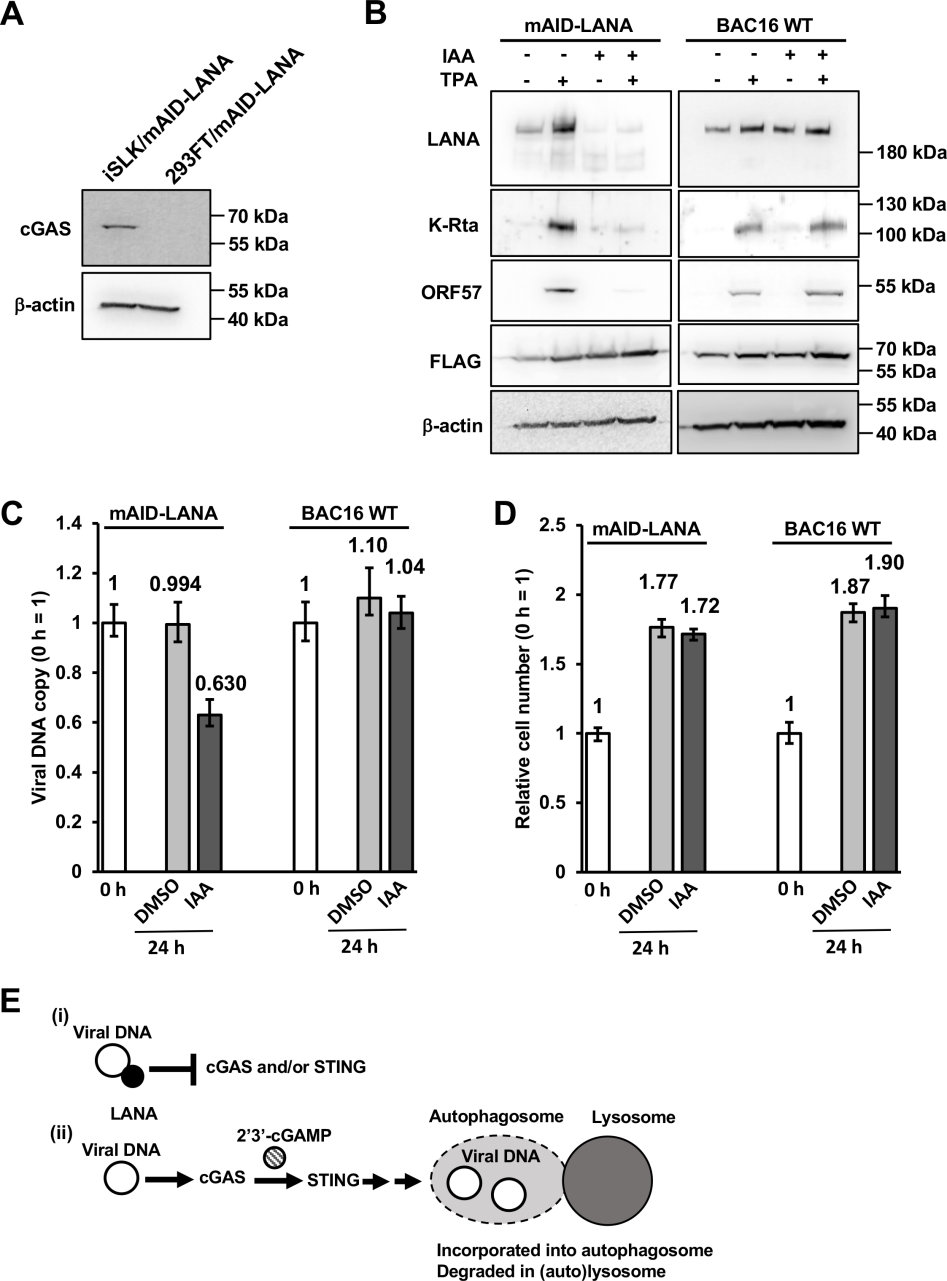
cGAS is required for the viral DNA reduction upon LANA depletion. (**A**) iSLK-OsTIR1-mAID-LANA BAC16 cells express cGAS but not in 293FT-OsTIR1-mAID-LANA BAC16 cells. Expression of cGAS was assessed by Western blotting. (**B**) The AID system is functional in 293FT cells. 293FT-OsTIR1-mAID-LANA BAC16 cells and 293FT-OsTIR1-BAC16 WT cells were treated with or without 2 μM of 5-Ph-IAA for 24 h, treated with or without 20 ng/mL of 12-*O*-tetradecanoylphorbol-13-acetate for another 24 h, and then the expression of viral proteins was assessed by Western blotting. (**C and D**) 293FT-OsTIR1-mAID-LANA BAC16 cells were treated with DMSO (0.1%) or 2-μM 5-Ph-IAA (IAA) for 24 h, and then followed by the indicated analyses. (**C**) Total DNA was extracted, and viral DNA copy was measured by real-time qPCR. 18S rRNA was used as an internal standard for normalization. (**D**) Cells were trypsinized and suspended in 1% BSA, 0.1-mM EDTA in PBS. The number of cells was manually counted by using a hemocytometer. The number of cells at 0 h was set as 1. (**E**) Hypothetical mechanism for how LANA protects viral DNA. (i) When LANA is present, LANA inhibits cGAS and/or STING to protect viral genomic DNA from degradation. (ii) When LANA is absent, cGAS produces 2′3′-cGAMP to stimulate STING. STING then induces autophagy. Viral genomic DNA is then delivered to the lysosome and degraded.

To further confirm if cGAS is required for viral DNA reduction, we employed a cGAS-null cell line. It is known that human embryonic kidney 293 cell and its sublineages lack the expression of cGAS (56). We first confirmed that iSLK-OsTIR1-mAID-LANA BAC16 cells express cGAS but not in 293FT-OsTIR1-mAID-LANA BAC16 cells (Fig. 5A). We then quantified viral DNA copy at 24 h after the addition of 5-Ph-IAA, and the results showed that viral DNA copy was only reduced by dilution during cell division in 293FT-OsTIR1-mAID-LANA BAC16 cells, suggesting that KSHV episome sensing by the cGAS is a critical step for episome degradation. We also performed the same set of experiments by using 293FT-OsTIR1-BAC16 WT cells and confirmed that observed phenotypes are due to LANA depletion (Fig. 5C and D). Next, the cells were treated with 5-Ph-IAA for 24 h, and lytic reactivation was induced with 20 ng/mL of 12-*O*-tetradecanoylphorbol-13-acetate (TPA) for another 24 h. As shown in Fig. 5B, LANA was depleted in response to 5-Ph-IAA. Interestingly, LANA depletion again strongly attenuated the expression of lytic proteins, K-Rta, and ORF57 to 12% of that of none-LANA depleted control with densitometry measurement. The results were similar to what we observed in iSLK-OsTIR1-mAID-LANA BAC16 cell, although in iSLK cells, expression of lytic proteins was further decreased to 5%. Non-linear association between KSHV episome copy numbers and lytic gene expression suggested that there might be a threshold that needs to meet for efficient lytic gene expression. The results suggested that degradation of episome is not the only reason for the attenuation of lytic gene transcriptions. Importantly, the depletion of LANA alone again did not trigger KSHV reactivation. With control, 293FT-OsTIR1-BAC16 WT cells, we confirmed that (i) the LANA depletion is AID dependent in 293FT cells, and (ii) 5-Ph-IAA treatment itself did not have effects on viral lytic gene expression induced by TPA treatment (Fig. 5B). Taken together, these results suggested that LANA is actively protecting the KSHV episome from the cGAS-STING axis (Fig. 5E).

## DISCUSSION

We applied the AID system to study the role of KSHV LANA in latency maintenance. By integrating a mAID-tag into KSHV BAC16 using a recombination-based method, we demonstrated the utility of this approach to study the function of a KSHV viral protein in infected cells. RNAi-mediated gene silencing has been utilized to study the function of specific genes/proteins of interest in various organisms in the past two decades. Some genes, however, cannot be knocked down because they are essential for cell viability. In addition, the RNAi approach relies on the turnover (half-life) of the target protein for efficient reduction in target protein levels. For silencing of most proteins, RNAi is usually effective only after 48–72 h of siRNA transfection (57). On the other hand, the inducible protein degradation approach allows us to quickly deplete the target protein (38, 39). Indeed, we showed that mAID-LANA was successfully depleted within 1.5 h after the addition of 5-Ph-IAA. We believe that the rapid depletion of target protein enables us to identify the biological effects more directly. After learning that mAID-LANA can be depleted rapidly and efficiently, we tagged several other viral proteins with mAID using the same recombination approach. We found that, however, K-Rta (ORF50) could not be depleted by treatment with 5-Ph-IAA in a similar time frame. In this method, the mAID-tagged target protein must be polyubiquitinated by SCF-OsTIR1 E3 ubiquitin ligase for proteasomal degradation. Accordingly, the abundance of target protein expression and accessibility of the E3 ligase to mAID-tag would have a significant impact on the outcome of target protein degradation. We speculate that having highly concentrated homo-multimers at specific chromosomal loci might be a reason for the great success of LANA degradation.

Previous studies revealed that LANA plays an important role in maintaining latency by repressing the transcriptional activity of the K-Rta promoter (45, 58). K-Rta is a key transcriptional regulator that controls the switch from latency to lytic replication (8). LANA downregulates K-Rta’s promoter activity in transient reporter assays, thus repressing K-Rta-mediated transactivation (59). LANA also physically interacts with K-Rta *in vitro* and in KSHV-infected cells (59). In addition, a study showed that KSHV can undergo spontaneous lytic reactivation, and K-Rta transcription level was increased when LANA expression was knocked down with a specific siRNA (60). These results establish a model in which LANA is actively suppressing viral lytic replication by antagonizing the functions of K-Rta. In the present study, we showed that the depletion of LANA itself did not induce lytic gene expression in iSLK cells. We found that LANA depletion instead further decreased viral lytic gene expression, which is triggered by doxycycline and sodium butyrate treatment. We noticed that KSHV latent chromatin is heavily modified with heterochromatin mark (H3K27me3) in iSLK cells when we compared with KSHV episomes in primary effusion lymphoma cells (61). This may partly explain why the depletion of LANA alone did not trigger reactivation. A detailed time course as well as increasing resolution of studies via isolation from reactivating from the dish would clarify LANA’s role in lytic gene transcription during reactivation.

Viral genomic copy number was reduced to approximately 25% of control within 24 h of 5-Ph-IAA incubation; the results strongly suggest that the KSHV episome is targeted by an active DNA degradation pathway in the absence of LANA. Sensing of viral constituents is the critical step in the host innate immune response against viruses (62). Several innate immune pathways have been identified, including TLRs (63), PRRs (64), and cGAS (65). Among the molecules involved in innate immunity, cGAS has emerged in recent years as a non-redundant dsDNA sensor important for detecting many pathogens (65). A recent study indeed demonstrated that LANA binds to cGAS and inhibits the cGAS-STING pathway and thereby antagonizes the cGAS-STING-mediated restriction of KSHV lytic replication (66). In the present study, we found that the viral genomic DNA degradation upon LANA depletion was mediated by the lysosome. In addition, cGAS, STING, and autophagy play a role in viral DNA degradation. Particularly, cGAS is required for the viral genomic DNA degradation in the absence of LANA (a model depicted in Fig. 5). A remaining question would be how does LANA antagonize cGAS to protect viral DNA? One possibility is that LANA binds to cGAS and prevents cGAS from recognizing dsDNA. However, this mechanism would require large copies of LANA molecules to absorb cGAS molecules neighboring to KSHV episomes. Locally concentrated LANA via TR binding and formation of nuclear bodies may therefore facilitate the function. For this, both LANA and cGAS are known to form liquid-liquid phase separations (67, 68). Our previous study also suggests that LANA may be involved in the formation of a three-dimensional (3D) episome structure through tight binding with TRs via a genomic looping (69). We speculate that rapid loss of LANA may therefore disrupt such 3D genomic structure, and this mechanism may facilitate the recognition of viral DNA by locally concentrated cGAS via LLPS. Further studies are required for clarifying how LANA plays a role in protecting latent viral episomes from degradation. Regardless of the specific mechanisms, this study again suggested that LANA is a very attractive target for therapeutic intervention for KSHV-associated diseases.

In summary, we successfully adapted the AID approach to recombinant KSHV BAC system and demonstrated the utility of the approach to assess viral protein function in infected cells. Our results proposed that LANA plays a critical role in preventing latent viral episomes from innate immune DNA sensing.

## MATERIALS AND METHODS

### Reagents

Dulbecco’s modified minimal essential medium (DMEM), fetal bovine serum (FBS), phosphate-buffered saline (PBS), trypsin-EDTA solution, 100× penicillin-streptomycin-L-glutamine solution (Pen-Strep-L-Gln), Alexa 405-conjugated secondary antibody, Alexa 555-conjugated secondary antibody, Alexa 647-conjugated secondary antibody, SlowFade Gold anti-fade reagent, Lipofectamine 2000 reagent, and high-capacity cDNA reverse transcription kit were purchased from Thermo Fisher (Waltham, MA, USA). Puromycin solution and Zeocin solution were obtained from InvivoGen (San Diego, CA, USA). Hygromycin B solution was purchased from Enzo Life Science (Farmingdale, NY, USA). Anti-FLAG M2 mouse monoclonal and anti-FLAG rabbit polyclonal antibodies, anti-LANA rat monoclonal antibody, anti-β-actin mouse monoclonal antibody, and polyvinylidene difluoride (PVDF) membrane were purchased from Millipore-Sigma (Burlington, MA, USA). Anti-K8α mouse monoclonal and anti-ORF57 mouse monoclonal antibodies were purchased from Santa Cruz Biotechnology (Santa Cruz, CA, USA). Anti-cGAS rabbit monoclonal antibody was purchased from Cell Signaling Technology (Danvers, MA, USA). Anti-mAID mouse monoclonal antibody was purchased from MBL (Tokyo, Japan). Anti-K-Rta rabbit polyclonal antibody was described previously (70). 5-Ph-IAA was purchased from MedChemExpress (Monmouth Junction, NJ, USA). cOmplete protease inhibitor cocktail tablets were purchased from Roche (Basel, Switzerland). ON-TARGETplus siRNAs were purchased from Horizon Discovery Ltd. (Cambridge, UK). The Quick-RNA Miniprep kit was purchased from Zymo Research (Irvine, CA, USA), and the QIAamp DNA mini kit was purchased from QIAGEN (Germantown, MD, USA). The NucleoBond Xtra BAC kit was purchased from TaKaRa Bio (Kusatsu, Shiga, Japan). All other chemicals were purchased from Millipore-Sigma or Fisher Scientific unless otherwise stated.

### Cell culture

iSLK cells were maintained in DMEM supplemented with 10% FBS, 1× Pen-Strep-L-Gln, and 2-μg/mL puromycin at 37°C with air containing 5% carbon dioxide. iSLK cell is a derivative of SLK cell, which was originally isolated from gingival endothelial tissue and was transduced with retroviruses expressing rtTA and K-Rta (71). 293FT cells were grown in DMEM supplemented with 10% FBS and 1× Pen-Strep-L-Gln at 37°C with air containing 5% carbon dioxide. iSLK.219 cells were maintained in DMEM supplemented with 10% FBS, 10-µg/mL puromycin, 400-µg/mL hygromycin B, and 250-µg/mL G418. iSLK cells harboring BAC16 WT were maintained in DMEM supplemented with 10% FBS, 1× Pen-Strep-L-Gln, 1,000-μg/mL hygromycin B, and 2-μg/mL puromycin at 37°C with air containing 5% carbon dioxide.

### Construction of mAID-LANA KSHV BAC16

Recombinant KSHV was prepared by following a protocol for *en passant* mutagenesis with a two-step markerless red recombination technique (46). Briefly, mAID-coding sequence was first synthesized (IDT DNA gBlock) and cloned into a pBlueScript SK vector. The sequence is indicated in Table 1. The pEPkan-S plasmid (72) was also used as a source of the kanamycin cassette, which includes an I-SceI restriction enzyme site at the 5′ end of the kanamycin resistance gene-coding region. The kanamycin cassette was amplified with primer pairs listed in Table 1. An amplified kanamycin cassette was then cloned into the mAID-coding region. The resulting plasmid was used as a template for another round of PCR to prepare a transfer DNA fragment for markerless recombination with BAC16. The PCR fragment was electroporated into *Escherichia coli* strain GS1783 harboring wild-type BAC16 using Bio-Rad *E. coli* Pulser. The electroporated *E. coli* was seeded onto a Luria broth (LB) agar plate containing 30-µg/mL chloramphenicol and 30-µg/mL kanamycin. Positive co-integrates were identified by colony PCR with appropriate primer pairs. Two independent co-integrates were cultured in LB containing 30-µg/mL chloramphenicol and 1% L-arabinose to induce I-SceI, and the *E. coli* was seeded onto an LB agar plate containing 30-µg/mL chloramphenicol and 1% L-arabinose. Positive clones were identified by colony PCR. The recombination junction and adjacent genomic regions were amplified by PCR, and the resulting PCR fragments were directly sequenced with the same primers to confirm in-frame insertion into the BAC DNA. Two independent BAC clones were generated as biological replicates. BAC DNA was extracted from *E. coli* using the NucleoBond Xtra BAC kit according to the manufacturer’s protocol. iSLK cells and 293FT cells seeded in a 10-cm dish were transfected with approximately 10 μg of purified mAID-LANA BAC16 using Lipofectamine 2000 reagent and selected with 1,000-μg/mL hygromycin B. iSLK cells and 293FT cells harboring mAID-LANA BAC16 were further transduced with recombinant lentivirus expressing FLAG-tagged OsTIR1 F74G protein and selected with-500 μg/mL Zeocin. The resulting cells were maintained in DMEM supplemented with 10% FBS, 1× Pen-Strep-L-Gln, 2-μg/mL puromycin, 1,000-μg/mL hygromycin B, and 100- to 200-μg/ml Zeocin.

### RNAi

iSLK-OsTIR1-mAID-LANA BAC16 cells were transfected with 25-nM siRNA against the indicated gene using Lipofectamine 2000 reagent. The cells were treated with or without 5-Ph-IAA for another 24 h, and then total DNA was extracted as described elsewhere.

### Western blotting

Cells were lysed in SUMO buffer consisting of 50-mM Tris-HCl (pH6.8), 1% SDS, 10% glycerol, and 1× protease inhibitor cocktail. Total cell lysates were boiled in SDS-PAGE sample buffer, subjected to SDS-PAGE, and subsequently transferred onto a PVDF membrane using a wet transfer apparatus (Bio-Rad, Hercules, CA, USA). The final dilutions of the primary antibodies were 1:500 for anti-LANA, 1:1,000 for anti-mAID, 1:2,500 for anti-K-Rta, 1:3,000 for anti-β-actin, 1:2,000 anti-FLAG (M2 mouse monoclonal), 1:200 for anti-ORF57, and 1:200 for anti-K8α. Membrane washes and secondary antibody incubations were performed as described previously (47).

### Real-time qPCR

Total RNA was isolated using a Quick-RNA Miniprep kit according to the manufacturer’s protocol. First-strand cDNA was synthesized using a high-capacity cDNA reverse transcription kit according to the manufacturer’s protocol. Gene expression was analyzed by real-time qPCR using specific primers for KSHV ORFs designed by Fakhari and Dittmer (73). We used 18S rRNA as an internal standard to normalize viral gene expression.

### Immunofluorescence microscopy

Cells grown on 22 × 22 mm glass coverslips were fixed with 4% paraformaldehyde in PBS for 20 min, permeabilized with 0.2% Triton X-100 in PBS for 20–30 min, and then blocked with 2% bovine serum albumin in PBS. The fixed cells were incubated with diluted primary antibody solution, followed by diluted secondary antibody solution. The cells on coverslips were then mounted on glass slides using SlowFade reagent and observed using a Keyence BX-Z710 fluorescence microscope (Osaka, Japan). The final dilutions of the primary antibodies were 1:100 for anti-LANA, 1:200 for anti-FLAG (rabbit polyclonal), and 1:100 for K8α.

### Flow cytometry

iSLK cells were infected with purified mAID-LANA virus at a MOI of 10. At approximately 48 h after infection, the cells were trypsinized, fixed with 2% paraformaldehyde, and suspended in 1% bovine serum albumin in PBS. Samples were acquired on Accuri C6 flow cytometer (BD Bioscience, Franklin Lakes, NJ, USA) and analyzed using FlowJo software (BD Bioscience) as described previously (47).

### Quantification of viral genomic DNA copy in cells

iSLK-OsTIR1-mAID-LANA BAC16 cells were seeded into six-well plates and treated with 2-μM 5-Ph-IAA or 0.1% dimethyl sulfoxide (DMSO) for 24 h. The cells were trypsinized and suspended in 200 μL of PBS. Total DNA was extracted using a QIAamp DNA mini kit according to the manufacturer’s protocol. Five microliters of eluate was used for real-time qPCR to determine viral genomic DNA copy number. For quantification of mitochondria DNA, we used a primer pair for tRNA Leu (UUR) that is encoded by mitochondria DNA (74). We used 18S rRNA as an internal standard to normalize viral genome copy numbers.

### Quantification of progeny virus production

iSLK-OsTIR1-mAID-LANA BAC16 cells were seeded into 6-well plates, and treated with 2 μM 5-Ph-IAA or 0.1% DMSO for 24 h. The cells were then treated with 1 μg/mL of doxycycline and 1.5-mM sodium butyrate for another 96 h together with 5-Ph-IAA. Two hundred microliters of cell culture supernatant was treated with 12 μg/mL DNase I for 15 min at room temperature to degrade DNAs that were not correctly encapsidated. The reaction was stopped by the addition of 5-mM EDTA, followed by heating at 70°C for 15 min. Viral DNA was then purified using a QIAamp DNA Mini kit according to the manufacturer’s protocol. Five microliters of eluate was used for real-time qPCR to determine viral genomic DNA copy number.

### Statistical analysis

Results are shown as means ± standard error of the mean from at least three independent experiments. Data were analyzed using an unpaired Student’s *t*-test. A false discovery rate (FDR)-corrected *P* value less than 0.05 was considered statistically significant.

## References

[1] Naimo E, Zischke J, Schulz TF. 2021. Recent advances in developing treatments of Kaposi’s sarcoma herpesvirus-related diseases. Viruses 13:1797. doi:10.3390/v13091797 34578378 PMC8473310

[2] Mesri EA, Feitelson MA, Munger K. 2014. Human viral oncogenesis: a cancer hallmarks analysis. Cell Host Microbe 15:266–282. doi:10.1016/j.chom.2014.02.011 24629334 PMC3992243

[3] Ganem D. 2007. KSHV-induced oncogenesis. In Arvin A, Campadelli-Fiume G, Mocarski E, Moore PS, Roizman B, Whitley R, Yamanishi K (ed), Human herpesviruses: biology, therapy, and immunoprophylaxis. Cambridge. 21348071

[4] Rivière L, Ducroux A, Buendia MA. 2014. The oncogenic role of hepatitis B virus. Recent Results Cancer Res 193:59–74. doi:10.1007/978-3-642-38965-8_4 24008293

[5] Szymonowicz KA, Chen J. 2020. Biological and clinical aspects of HPV-related cancers. Cancer Biol Med 17:864–878. doi:10.20892/j.issn.2095-3941.2020.0370 33299640 PMC7721094

[6] Herbein G. 2018. The human cytomegalovirus, from oncomodulation to oncogenesis. Viruses 10:408. doi:10.3390/v10080408 30081496 PMC6115842

[7] Cai Q, Verma SC, Lu J, Robertson ES. 2010. Molecular biology of Kaposi's sarcoma-associated herpesvirus and related oncogenesis. Adv Virus Res 78:87–142. doi:10.1016/B978-0-12-385032-4.00003-3 21040832 PMC3142360

[8] Purushothaman P, Uppal T, Verma SC. 2015. Molecular biology of KSHV lytic reactivation. Viruses 7:116–153. doi:10.3390/v7010116 25594835 PMC4306831

[9] Dissinger NJ, Damania B. 2016. Recent advances in understanding Kaposi's sarcoma-associated herpesvirus. F1000Res 5:F1000 Faculty Rev-740. doi:10.12688/f1000research.7612.1 PMC4847565 27158465

[10] Aneja KK, Yuan Y. 2017. Reactivation and lytic replication of Kaposi’s sarcoma-associated herpesvirus: an update. Front Microbiol 8:613. doi:10.3389/fmicb.2017.00613 28473805 PMC5397509

[11] Collins CM, Medveczky PG. 2002. Genetic requirements for the episomal maintenance of oncogenic herpesvirus genomes. Adv Cancer Res 84:155–174. doi:10.1016/s0065-230x(02)84005-2 11883526

[12] Domsic JF, Chen HS, Lu F, Marmorstein R, Lieberman PM. 2013. Molecular basis for oligomeric-DNA binding and episome maintenance by KSHV LANA. PLoS Pathog 9:e1003672. doi:10.1371/journal.ppat.1003672 24146617 PMC3798644

[13] Purushothaman P, Dabral P, Gupta N, Sarkar R, Verma SC. 2016. KSHV genome replication and maintenance. Front Microbiol 7:54. doi:10.3389/fmicb.2016.00054 26870016 PMC4740845

[14] Uppal T, Banerjee S, Sun Z, Verma SC, Robertson ES. 2014. KSHV LANA--the master regulator of KSHV latency. Viruses 6:4961–4998. doi:10.3390/v6124961 25514370 PMC4276939

[15] Cotter MA, Robertson ES. 1999. The latency-associated nuclear antigen tethers the Kaposi's sarcoma-associated herpesvirus genome to host chromosomes in body cavity-based lymphoma cells. Virology 264:254–264. doi:10.1006/viro.1999.9999 10562490

[16] Van Dross R, Yao S, Asad S, Westlake G, Mays DJ, Barquero L, Duell S, Pietenpol JA, Browning PJ. 2005. Constitutively active K-cyclin/cdk6 kinase in Kaposi sarcoma-associated herpesvirus-infected cells. J Natl Cancer Inst 97:656–666. doi:10.1093/jnci/dji113 15870436

[17] Guasparri I, Keller SA, Cesarman E. 2004. KSHV vFLIP is essential for the survival of infected lymphoma cells. J Exp Med 199:993–1003. doi:10.1084/jem.20031467 15067035 PMC2211879

[18] Sadler R, Wu L, Forghani B, Renne R, Zhong W, Herndier B, Ganem D. 1999. A complex translational program generates multiple novel proteins from the latently expressed kaposin (K12) locus of Kaposi's sarcoma-associated herpesvirus. J Virol 73:5722–5730. doi:10.1128/JVI.73.7.5722-5730.1999 10364323 PMC112632

[19] Verma SC, Choudhuri T, Kaul R, Robertson ES. 2006. Latency-associated nuclear antigen (LANA) of Kaposi's sarcoma-associated herpesvirus interacts with origin recognition complexes at the LANA binding sequence within the terminal repeats. J Virol 80:2243–2256. doi:10.1128/JVI.80.5.2243-2256.2006 16474132 PMC1395374

[20] Rahayu R, Ohsaki E, Omori H, Ueda K. 2016. Localization of latency-associated nuclear antigen (LANA) on mitotic chromosomes. Virology 496:51–58. doi:10.1016/j.virol.2016.05.020 27254595

[21] Ballestas ME, Chatis PA, Kaye KM. 1999. Efficient persistence of extrachromosomal KSHV DNA mediated by latency-associated nuclear antigen. Science 284:641–644. doi:10.1126/science.284.5414.641 10213686

[22] Shinohara H, Fukushi M, Higuchi M, Oie M, Hoshi O, Ushiki T, Hayashi J-I, Fujii M. 2002. Chromosome binding site of latency-associated nuclear antigen of Kaposi's sarcoma-associated herpesvirus is essential for persistent episome maintenance and is functionally replaced by histone H1. J Virol 76:12917–12924. doi:10.1128/jvi.76.24.12917-12924.2002 12438617 PMC136661

[23] Verma SC, Cai Q, Kreider E, Lu J, Robertson ES. 2013. Comprehensive analysis of LANA interacting proteins essential for viral genome tethering and persistence. PLoS One 8:e74662. doi:10.1371/journal.pone.0074662 24040311 PMC3770571

[24] Chen H-S, De Leo A, Wang Z, Kerekovic A, Hills R, Lieberman PM, Dittmer DP. 2017. BET-inhibitors disrupt Rad21-dependent conformational control of KSHV latency. PLoS Pathog 13:e1006100. doi:10.1371/journal.ppat.1006100 28107481 PMC5287475

[25] Ottinger M, Christalla T, Nathan K, Brinkmann MM, Viejo-Borbolla A, Schulz TF. 2006. Kaposi's sarcoma-associated herpesvirus LANA-1 interacts with the short variant of BRD4 and releases cells from a BRD4- and BRD2/RING3-induced G1 cell cycle arrest. J Virol 80:10772–10786. doi:10.1128/JVI.00804-06 16928766 PMC1641788

[26] Viejo-Borbolla A, Ottinger M, Brüning E, Bürger A, König R, Kati E, Sheldon JA, Schulz TF. 2005. Brd2/RING3 interacts with a chromatin-binding domain in the Kaposi's sarcoma-associated herpesvirus latency-associated nuclear antigen 1 (LANA-1) that is required for multiple functions of LANA-1. J Virol 79:13618–13629. doi:10.1128/JVI.79.21.13618-13629.2005 16227282 PMC1262589

[27] Hellert J, Weidner-Glunde M, Krausze J, Richter U, Adler H, Fedorov R, Pietrek M, Rückert J, Ritter C, Schulz TF, Lührs T. 2013. A structural basis for BRD2/4-mediated host chromatin interaction and oligomer assembly of Kaposi sarcoma-associated herpesvirus and murine gammaherpesvirus LANA proteins. PLoS Pathog 9:e1003640. doi:10.1371/journal.ppat.1003640 24146614 PMC3798688

[28] Kim KY, Huerta SB, Izumiya C, Wang DH, Martinez A, Shevchenko B, Kung HJ, Campbell M, Izumiya Y. 2013. Kaposi's sarcoma-associated herpesvirus (KSHV) latency-associated nuclear antigen regulates the KSHV epigenome by association with the histone demethylase KDM3A. J Virol 87:6782–6793. doi:10.1128/JVI.00011-13 23576503 PMC3676133

[29] Hu J, Yang Y, Turner PC, Jain V, McIntyre LM, Renne R. 2014. LANA binds to multiple active viral and cellular promoters and associates with the H3K4methyltransferase hSET1 complex. PLoS Pathog 10:e1004240. doi:10.1371/journal.ppat.1004240 25033463 PMC4102568

[30] Toth Z, Papp B, Brulois K, Choi YJ, Gao SJ, Jung JU. 2016. LANA-mediated recruitment of host polycomb repressive complexes onto the KSHV genome during de novo infection. PLoS Pathog 12:e1005878. doi:10.1371/journal.ppat.1005878 27606464 PMC5015872

[31] Tan M, Li S, Juillard F, Chitas R, Custódio TF, Xue H, Szymula A, Sun Q, Liu B, Álvarez ÁL, Chen S, Huang J, Simas JP, McVey CE, Kaye KM. 2021. MLL1 is regulated by KSHV LANA and is important for virus latency. Nucleic Acids Res 49:12895–12911. doi:10.1093/nar/gkab1094 34850113 PMC8682764

[32] Kumar A, Lyu Y, Yanagihashi Y, Chantarasrivong C, Majerciak V, Salemi M, Wang KH, Inagaki T, Chuang F, Davis RR, Tepper CG, Nakano K, Izumiya C, Shimoda M, Nakajima KI, Merleev A, Zheng ZM, Campbell M, Izumiya Y. 2022. KSHV episome tethering sites on host chromosomes and regulation of latency-lytic switch by CHD4. Cell Rep 39:110788. doi:10.1016/j.celrep.2022.110788 35545047 PMC9153692

[33] Di C, Zheng G, Zhang Y, Tong E, Ren Y, Hong Y, Song Y, Chen R, Tan X, Yang L. 2021. RTA and LANA competitively regulate let-7A/RBPJ signal to control KSHV replication. Front Microbiol 12:804215. doi:10.3389/fmicb.2021.804215 35069510 PMC8777081

[34] Günther T, Grundhoff A, Kellam P. 2010. The epigenetic landscape of latent Kaposi sarcoma-associated herpesvirus genomes. PLoS Pathog 6:e1000935. doi:10.1371/journal.ppat.1000935 20532208 PMC2880564

[35] Toth Z, Maglinte DT, Lee SH, Lee HR, Wong LY, Brulois KF, Lee S, Buckley JD, Laird PW, Marquez VE, Jung JU. 2010. Epigenetic analysis of KSHV latent and lytic genomes. PLoS Pathog 6:e1001013. doi:10.1371/journal.ppat.1001013 20661424 PMC2908616

[36] Toth Z, Smindak RJ, Papp B. 2017. Inhibition of the lytic cycle of Kaposi's sarcoma-associated herpesvirus by cohesin factors following de novo infection. Virology 512:25–33. doi:10.1016/j.virol.2017.09.001 28898712 PMC5653454

[37] Röth S, Fulcher LJ, Sapkota GP. 2019. Advances in targeted degradation of endogenous proteins. Cell Mol Life Sci 76:2761–2777. doi:10.1007/s00018-019-03112-6 31030225 PMC6588652

[38] Nishimura K, Fukagawa T, Takisawa H, Kakimoto T, Kanemaki M. 2009. An auxin-based degron system for the rapid depletion of proteins in nonplant cells. Nat Methods 6:917–922. doi:10.1038/nmeth.1401 19915560

[39] Yesbolatova A, Saito Y, Kitamoto N, Makino-Itou H, Ajima R, Nakano R, Nakaoka H, Fukui K, Gamo K, Tominari Y, Takeuchi H, Saga Y, Hayashi KI, Kanemaki MT. 2020. The auxin-inducible degron 2 technology provides sharp degradation control in yeast, mammalian cells, and mice. Nat Commun 11:5701. doi:10.1038/s41467-020-19532-z 33177522 PMC7659001

[40] Buchmann K. 2014. Evolution of innate immunity: clues from invertebrates via fish to mammals. Front Immunol 5:459. doi:10.3389/fimmu.2014.00459 25295041 PMC4172062

[41] Bryant CE, Monie TP. 2012. Mice, men and the relatives: cross-species studies underpin innate immunity. Open Biol 2:120015. doi:10.1098/rsob.120015 22724060 PMC3376732

[42] Mogensen TH. 2009. Pathogen recognition and inflammatory signaling in innate immune defenses. Clin Microbiol Rev 22:240–273. doi:10.1128/CMR.00046-08 19366914 PMC2668232

[43] Sun Z, Hornung V. 2022. cGAS-STING signaling. Curr Biol 32:R730–R734. doi:10.1016/j.cub.2022.05.027 35820380

[44] Gui X, Yang H, Li T, Tan X, Shi P, Li M, Du F, Chen ZJ. 2019. Autophagy induction via STING trafficking is a primordial function of the cGAS pathway. Nature 567:262–266. doi:10.1038/s41586-019-1006-9 30842662 PMC9417302

[45] Qi Y, Zheng G, Di C, Zhang J, Wang X, Hong Y, Song Y, Chen R, Yang Y, Yan Y, Xu L, Tan X, Yang L. 2019. Latency-associated nuclear antigen inhibits lytic replication of Kaposi’s sarcoma-associated herpesvirus by regulating Let-7A/RBPJ signaling. Virology 531:69–78. doi:10.1016/j.virol.2019.02.019 30856484

[46] Tischer BK, Smith GA, Osterrieder N. 2010. En passant mutagenesis: a two step markerless red recombination system. Methods Mol Biol 634:421–430. doi:10.1007/978-1-60761-652-8_30 20677001

[47] Nakajima K-I, Guevara-Plunkett S, Chuang F, Wang KH, Lyu Y, Kumar A, Luxardi G, Izumiya C, Soulika A, Campbell M, Izumiya Y. 2020. Rainbow Kaposi's sarcoma-associated Herpesvirus revealed Heterogenic replication with dynamic gene expression. J Virol 94:e01565-19. doi:10.1128/JVI.01565-19 31969436 PMC7108829

[48] Kumar A, Salemi M, Bhullar R, Guevara-Plunkett S, Lyu Y, Wang KH, Izumiya C, Campbell M, Nakajima KI, Izumiya Y. 2021. Proximity biotin labeling reveals Kaposi’s sarcoma-associated herpesvirus interferon regulatory factor networks. J Virol 95:e02049-20. doi:10.1128/JVI.02049-20 33597212 PMC8104114

[49] De Leo A, Deng Z, Vladimirova O, Chen H-S, Dheekollu J, Calderon A, Myers KA, Hayden J, Keeney F, Kaufer BB, Yuan Y, Robertson E, Lieberman PM, Kaye KM. 2019. LANA oligomeric architecture is essential for KSHV nuclear body formation and viral genome maintenance during latency. PLoS Pathog 15:e1007489. doi:10.1371/journal.ppat.1007489 30682185 PMC6364946

[50] Xu H, Ren D. 2015. Lysosomal physiology. Annu Rev Physiol 77:57–80. doi:10.1146/annurev-physiol-021014-071649 25668017 PMC4524569

[51] Yim W-Y, Mizushima N. 2020. Lysosome biology in autophagy. Cell Discov 6:6. doi:10.1038/s41421-020-0141-7 32047650 PMC7010707

[52] Mindell JA. 2012. Lysosomal acidification mechanisms. Annu Rev Physiol 74:69–86. doi:10.1146/annurev-physiol-012110-142317 22335796

[53] Winzeler EA. 2008. Malaria research in the post-genomic era. Nature 455:751–756. doi:10.1038/nature07361 18843360 PMC2705782

[54] Bonam SR, Wang F, Muller S. 2019. Lysosomes as a therapeutic target. Nat Rev Drug Discov 18:923–948. doi:10.1038/s41573-019-0036-1 31477883 PMC7097195

[55] Zhu Y, An X, Zhang X, Qiao Y, Zheng T, Li X. 2019. STING: a master regulator in the cancer-immunity cycle. Mol Cancer 18:152. doi:10.1186/s12943-019-1087-y 31679519 PMC6827255

[56] Sun L, Wu J, Du F, Chen X, Chen ZJ. 2013. Cyclic GMP-AMP synthase is a cytosolic DNA sensor that activates the type I interferon pathway. Science 339:786–791. doi:10.1126/science.1232458 23258413 PMC3863629

[57] Krueger U, Bergauer T, Kaufmann B, Wolter I, Pilk S, Heider-Fabian M, Kirch S, Artz-Oppitz C, Isselhorst M, Konrad J. 2007. Insights into effective RNAi gained from large-scale siRNA validation screening. Oligonucleotides 17:237–250. doi:10.1089/oli.2006.0065 17638527

[58] Broussard G, Damania B. 2020. Regulation of KSHV latency and lytic reactivation. Viruses 12:1034. doi:10.3390/v12091034 32957532 PMC7551196

[59] Lan K, Kuppers DA, Verma SC, Robertson ES. 2004. Kaposi’s sarcoma-associated herpesvirus-encoded latency-associated nuclear antigen inhibits lytic replication by targeting RTA: A potential mechanism for virus-mediated control of latency. J Virol 78:6585–6594. doi:10.1128/JVI.78.12.6585-6594.2004 15163750 PMC416549

[60] Godfrey A, Anderson J, Papanastasiou A, Takeuchi Y, Boshoff C. 2005. Inhibiting primary effusion lymphoma by lentiviral vectors encoding short hairpin RNA. Blood 105:2510–2518. doi:10.1182/blood-2004-08-3052 15572586

[61] Izumiya Y, Algalil A, Espera JM, Miura H, Izumiya C, Inagaki T, Kumar A. 2024. Kaposi's sarcoma-associated herpesvirus terminal repeat regulates inducible lytic gene promoters. J Virol:e01386-23. doi:10.1128/jvi.01386-23 PMC10878276 38240593

[62] Paludan SR, Pradeu T, Masters SL, Mogensen TH. 2021. Constitutive immune mechanisms: mediators of host defence and immune regulation. Nat Rev Immunol 21:137–150. doi:10.1038/s41577-020-0391-5 32782357 PMC7418297

[63] Fitzgerald KA, Kagan JC. 2020. Toll-like receptors and the control of immunity. Cell 180:1044–1066. doi:10.1016/j.cell.2020.02.041 32164908 PMC9358771

[64] Gulati A, Kaur D, Krishna Prasad GVR, Mukhopadhaya A. 2018. PRR function of innate immune receptors in recognition of bacteria or bacterial ligands. Adv Exp Med Biol 1112:255–280. doi:10.1007/978-981-13-3065-0_18 30637703

[65] Tao J, Zhou X, Jiang Z. 2016. cGAS-cGAMP-STING: The three musketeers of cytosolic DNA sensing and signaling. IUBMB Life 68:858–870. doi:10.1002/iub.1566 27706894

[66] Zhang G, Chan B, Samarina N, Abere B, Weidner-Glunde M, Buch A, Pich A, Brinkmann MM, Schulz TF. 2016. Cytoplasmic isoforms of Kaposi sarcoma herpesvirus LANA recruit and antagonize the innate immune DNA sensor cGAS. Proc Natl Acad Sci U S A 113:E1034–E1043. doi:10.1073/pnas.1516812113 26811480 PMC4776510

[67] Vladimirova O, De Leo A, Deng Z, Wiedmer A, Hayden J, Lieberman PM. 2021. Phase separation and DAXX redistribution contribute to LANA nuclear body and KSHV genome dynamics during latency and reactivation. PLoS Pathog 17:e1009231. doi:10.1371/journal.ppat.1009231 33471863 PMC7943007

[68] Du M, Chen ZJ. 2018. DNA-induced liquid phase condensation of cGAS activates innate immune signaling. Science 361:704–709. doi:10.1126/science.aat1022 29976794 PMC9417938

[69] Campbell M, Chantarasrivong C, Yanagihashi Y, Inagaki T, Davis RR, Nakano K, Kumar A, Tepper CG, Izumiya Y, Jung JU. 2022. KSHV topologically associating domains in latent and reactivated viral chromatin. J Virol 96:e0056522. doi:10.1128/jvi.00565-22 35867573 PMC9327698

[70] Izumiya Y, Izumiya C, Van Geelen A, Wang D-H, Lam KS, Luciw PA, Kung H-J. 2007. Kaposi's sarcoma-associated herpesvirus-encoded protein kinase and its interaction with K-bZIP. J Virol 81:1072–1082. doi:10.1128/JVI.01473-06 17108053 PMC1797516

[71] Myoung J, Ganem D. 2011. Generation of a doxycycline-inducible KSHV producer cell line of endothelial origin: maintenance of tight latency with efficient reactivation upon induction. J Virol Methods 174:12–21. doi:10.1016/j.jviromet.2011.03.012 21419799 PMC3095772

[72] Tischer BK, von Einem J, Kaufer B, Osterrieder N. 2006. Two-step red-mediated recombination for versatile high-efficiency markerless DNA manipulation in Escherichia coli. Biotechniques 40:191–197. doi:10.2144/000112096 16526409

[73] Fakhari FD, Dittmer DP. 2002. Charting latency transcripts in Kaposi's sarcoma-associated herpesvirus by whole-genome real-time quantitative PCR. J Virol 76:6213–6223. doi:10.1128/jvi.76.12.6213-6223.2002 12021355 PMC136228

[74] Rooney JP, Ryde IT, Sanders LH, Howlett EH, Colton MD, Germ KE, Mayer GD, Greenamyre JT, Meyer JN. 2015. PCR based determination of mitochondrial DNA copy number in multiple species. Methods Mol Biol 1241:23–38. doi:10.1007/978-1-4939-1875-1_3 25308485 PMC4312664

